# Recovery and Utilization of Pea Albumins as Acidic Emulsion Stabilizer by Complexation with Dextran Sulfate

**DOI:** 10.3390/foods11233784

**Published:** 2022-11-24

**Authors:** Xingfei Li, Xinyu Zhang, Jie Long, Caimeng Zhang, Yufei Hua

**Affiliations:** State Key Laboratory of Food Science and Technology, School of Food Science and Technology, Collaborative Innovation Center of Food Safety and Quality Control in Jiangsu Province, Jiangnan University, 1800 Lihu Avenue, Wuxi 214122, China

**Keywords:** pea albumins, dextran sulfate, complexation, heating, interface adsorption

## Abstract

In this work, pea albumins (PAs) were efficiently recovered by complexation with dextran sulfate (DS), and the emulsifying ability and stability of PA/DS complexes were studied. The largest amounts of PAs (81.25%) were recovered at *r* = 5:1 and pH_max_ (pH 3.41) by forming insoluble complexes; and only soluble complexes were formed at *r* = 2:1 and over the whole pH range (2.0–7.0). The emulsions stabilized by PA/DS soluble complexes remained stable under acidic conditions due to the highly negatively charge (from −45.10 ± 0.40 to −57.23 ± 0.66 mV) and small particle size (0.168 ± 0.010–0.448 ± 0.004 μm), while emulsions stabilized by PAs alone generated a strong creaming and serum separation at pH 5 and 6. In terms of emulsifying stability, all PA emulsions and unheated PA/DS emulsions became unstable with different creaming index after 14 days storage. SDS-PAGE results showed that the interface adsorption proteins of unheated emulsions mainly consisted of PA1a, which was unfavorable to the stability of the interface. On the contrary, heat treatment (95 °C, 30 min) and complexation (PA/DS = 2:1) enhanced the adsorption of PA2 and lectin at the interface, inhibiting the aggregation of PA2 and lectin. This resulted in long-term stability of the PA/DS emulsions under acidic conditions.

## 1. Introduction

Pea albumins (PAs) account for approximately 15–25% of the total pea seed proteins. They are well known for their high nutritional value because they are rich in essential amino acids, especially sulfur amino acids [1,2]. Pea albumins mainly consist of three components: lectin, pea albumin 1 (PA1), and pea albumin 2 (PA2). Lectin has a molecular weight of 50 kDa and consists of two subunits, A and B, with a molecular weight of 6 and 19 kDa, respectively. Lectin specifically recognizes mannose and glucose and is known to have interesting pharmacological activities [3,4,5,6]. PA1 contains both PA1a (Mw 6000 Da) and PA1b (Mw 4000 Da) peptides, and contributes ~50% of the sulphur amino acids in the seed. PA2, a homodimer of two polypeptides (PA2a and PA2b) of approximately 25–26 kDa, accounts for about 16% of the total sulphur amino acids [7,8]. PA1 has been proved to have insecticidal effects [9]. Mariotti et al. [10] reported that PAs can improve the postprandial biological value of pea protein. Chudzik-Kozłowska et al. [11] reported that the immunomodulatory properties of PAs and glycated PAs, and the lack of allergenic potential, renders them suitable for supplements in personalized diets.

Pea albumins are predominantly found in the pea whey, which is largely generated as by-products of wet processes for pea protein production [12]. Based on the storage proteins’ composition of various plant sources, it was estimated that about 10–40 g of albumin was produced for each 100 g of globulins extracted [13]. However, the albumins are recovered with low efficiency, and the direct discharge of them as waste poses a major environmental issue [14]. Gao et al. [15] studied the recovery of proteins from a pea whey by ultrafiltration, and the protein yield was 15 to 53 g/100 g of total pea proteins. Protein-polysaccharide interaction is another potential strategy for the recovery and improvement of the functional properties of proteins [16], etc. In our previous work, PAs were recovered by complexation with cationic polysaccharide, but with a low recovery rate (<40%) [17].

The functional properties of PAs have attracted more attention in recent years, including their emulsifying, gelling properties [18], and foaming abilities. The good solubility of PAs under acidic conditions provides the possibility for developing an acid emulsion stabilizer. The albumin-rich fraction was found to be less effective in oil droplet stabilization and less resistant to oil droplet flocculation, as compared to the globulin-rich fraction [19]. Lu et al. [20] demonstrated that the functionality of albumins was the highest at acidic pH, and the presence of PA2 albumin resulted in the best foaming and emulsifying properties. Luo et al. [21] detailed studied the adsorption kinetics and dilatational rheological properties of recombinant PA2, and their results revealed that the adsorption of PA2 at the oil–water interface was mainly concentration-dependent, and in the form of protein aggregates. However, the emulsifying ability of PAs under acidic conditions and the possible competitive adsorption behavior among PA2, PA1, and lectin have not yet been systematically examined.

In this paper, PAs were first recovered using a strong anionic polysaccharide by forming strong electrostatic complexation with proteins, and their complex behavior, protein recovery and protein compositions were analyzed. We hypothesized that PA/DS complexes and PAs alone exhibited different emulsifying properties, because their molecular properties, including size, hydrophobicity, and net charge, were different. The emulsifying ability of PA/DS complexes was studied under acidic conditions in terms of interfacial adsorption behavior, zeta-potential and particle size of the emulsion, morphology, apparent viscosity, interfacial adsorption protein composition, and creaming index. This study provides insights into the emulsifying ability and interface adsorption behavior of PAs as influenced by their complexation behavior.

## 2. Materials and Methods

### 2.1. Materials

Yellow pea (*Pisum sativum* L.) seeds were provided by Yantai Shuangta Food Co., Ltd. (Yantai, Shandong, China). Sunflower oil was provided by the local supermarket (Wuxi, China). N,N′-methyldiacrylamide, acrylamide, tetraethyl ethylenediamine (TEMED), Fluorescein isothiocyanate (FITC), dextran sulfate (DS, MW 500 kDa, 16.0–19.0% sulfate content), and Nile red were provided by Sigma-Aldrich (Shanghai, China). Hydrochloric acid, sodium hydroxide, bicinchoninic acid, ammonium sulfate, gel eletrophoresis reagents chemicals including sodium dodecyl sulfate (SDS), trihydroxymethylaminomethane (Tris), dithiothreitol (DTT), glycine, trichloroacetic acid, ammonium persulfate, and Coomassie Brilliant Blue G-250, were provided by Sinopharm Chemical Reagent Co., Ltd. (Shanghai, China).

### 2.2. Preparation of Pea Whey

Pea whey was prepared according to Yang et al. [17]. Specifically, pea seeds were ground in a grinder (QE-200, Yili Industry and Trade Co., Ltd., Zhejiang, China) for 2.0 min, and the obtained pea meal was defatted with hexane (1:3, *w*/*v*) and dried overnight in a fume hood. Then, 300 g of defatted pea flour was mixed with 3.0 L deionized water (*w*/*v*, 1:10), and adjusted to the pH 9.0 using 2.0 M NaOH. After kept stirring for 1 h, the suspension was centrifuged at 9000 rpm for 30 min to remove insoluble residue. The obtained supernatant was adjusted to pH 4.5 using 2.0 M HCl and then centrifuged at 9000 rpm for 30 min. After removing the protein precipitates, the supernatant was collected and kept overnight at 4 °C before further centrifugation (9000 rpm, 30 min) to remove the residual insoluble components. The prepared pea whey (protein concentration ~0.43% *w*/*v*) was stored at 4 °C by adding 0.02% sodium azide to prevent microbial growth before further use.

### 2.3. Recovery of PAs by Complexation with Dextran Sulfate

A solution of dextran sulfate (DS) (0.43% (*w*/*v*)) was prepared by dissolving 0.43 g of DS powder in 100 mL deionized water and stirring for 3 h. Mixtures of pea whey and DS solutions with desired mass ratios (2:1–20:1) were titrated with different concentrations (0.1–2.0 M) of HCl to the pH 2.0, and the absorbance at 600 nm was recorded as a function of pH. The complexes obtained at pH_max_ were further centrifuged at 4000 rpm for 15 min to recover the precipitates and supernatant. The protein recovery was calculated from the protein content of supernatant determined by bicinchoninic acid (BCA) method (Smith et al., 1985).

### 2.4. Preparation of PA/DS Soluble Complexes and PAs

PAs were recovered at PA/DS = 5:1, pH 3.41 (pH_max_), and the precipitates were collected by centrifugation at 4000 rpm for 15 min. The precipitates were dispersed at a certain volume of deionized water and then adjusted to pH 7 to dissolve the biopolymers. Subsequently, a certain amount of DS powder was added to reach a final protein/polysaccharide mass ratio of 2:1. The above solutions were diluted to a final protein concentration of 1.0% (*w*/*v*) using deionized water. Then, the PA/DS soluble complexes were adjusted to a pH of 7.0, 6.0, 5.0, and 4.0 using 2.0 M HCl.

As a control, PAs were recovered using the ammonium sulfate method. Briefly, a certain amount of ammonium sulfate was gradually added to the 1.0 L of pea whey to reach 80% saturation, with gentle stirring at room temperature. Then, the pea whey was centrifuged at 9000 rpm for 30 min to collect the precipitates. The precipitates were dispersed at a certain volume of deionized water and adjusted to pH 7 to dissolve the proteins. The crude protein solutions were dialyzed for 48 h against deionized water, and then freeze-dried at −40 °C for 48 h to obtain the PAs powder. The prepared PAs powder contained 96.91 ± 0.06% protein, 2.25 ± 0.04% ash, and 0.84 ± 0.02% carbohydrate (based on dry basis), and the protein component (PA1, PA2 and lectin) of PAs was similar to that recovered by complexation with dextran sulfate (PA1, PA2 and lectin.

### 2.5. Interfacial Tension Measurements

The interfacial tensions of PAs and PA/DS solutions as a function of pH were determined with video optical contact angle analyzer (OCA 15 EC, Data Physics Instruments GmbH, Filderstadt, Germany) using the pendant drop method [22,23]. About 10 μL of PAs or PA/DS solution drop on the needle tip was automatically injected into cylinder containing sunflower oil, and the optical image of the droplet was recorded and analyzed to obtain σ. The interfacial pressure (π) could be calculated as:

π = σ_0_ − σ (1)

where σ_0_ and σ are the interfacial tension of distilled water and PA/DS or PA solution, respectively. The change of surface pressure (*π*) as a function of adsorption time *t* is expressed as Equation (2) [24], and the diffusion rate constant (*K*_dif_) was calculated from the slope of the linear part of the *π*-t^1/2^ curve.

*π* = 2*C_0_KT*(*Dt*/3.14)^1/2^ (2)

where *C*_0_ is the protein concentration, *K* is the Boltzmann constant, *D* is the diffusion coefficient, *T* is the absolute temperature, and *t* is the adsorption time.

### 2.6. Emulsion Preparation

To prepare PA/DS emulsions, the PA/DS soluble complexes solution containing 1.0% (*w*/*v*) protein was firstly prepared following the procedure of method 2.4. Then, the above PA/DS solution was mixed with sunflower seed oil (9:1, *v*/*v*) and homogenized at 10,000 rpm for 90 s with a high-speed homogenizer (FA25 model, Shanghai, China). Subsequently, the emulsions were further homogenized with a high-pressure homogenizer (AH2010, Shanghai, China) for another 90 s at 60 MPa to prepare the freshly prepared unheated PA/DS emulsions. As a control, the freshly prepared emulsion was further heated at 95 °C for 30 min to prepare heated PA/DS emulsions. Sodium azide with 0.02% (*w*/*v*) was added to the emulsions to prevent microbial growth.

To prepare PA emulsions, PA powder was firstly prepared following the procedure of method 2.4. Then, about 1.03 g of PAs powder was dissolved in the 100 mL of deionized water to obtain a final protein concentration of 1.0% (*w*/*v*). The PAs solution was further mixed with sunflower seed oil (9:1, *v*/*v*) and homogenized at 10,000 rpm for 90 s with a high-speed homogenizer (FA25 model, Shanghai, China). Afterwards, the unheated and heated PA emulsions were prepared following the same procedure as mentioned above for the PA/DS emulsions.

### 2.7. Determination of Zeta Potential and Oil Droplet Size

Zeta potentials of the PA or PA/DS emulsions were measured according to the method described by Zhang et al. [25] with some modifications. The experiment was carried out with Nano-ZS Zetasizer (Malvern Instruments, Malvern, UK). Before measurements, the protein emulsion was diluted to the final protein concentration of 0.01% (*w*/*v*). Measurements were tested three times at 25 °C.

The mean particle sizes of the PAs or PA/DS emulsions were determined with a laser particle size analyzer (Microtrac S3500, Montgomeryville, USA). The mean particle sizes were presented as volume average particle size (d_4, 3_).

### 2.8. Apparent Viscosity

The apparent viscosity as a function of the shear rate (0–200 s^−1^) of the PA or PA/DS emulsions was determined with a rheometer (Anton MCR301, Graz, Austria). A plate probe (PP50, 50 mm × 1 mm) was used. Measurements were carried out in triplicate at 25 °C.

### 2.9. Confocal Laser Scanning Microscopy (CLSM)

The morphologies of freshly prepared PA and PA/DS emulsions were characterized by confocal laser scanning microscopy (CLSM, TCS SP8, Leica, Heidelberg, Germany). Before measurements, the emulsion was separately stained with fluorescein isothiocyanate (FITC, 0.05%, *w*/*v*) and Nile Red (0.05%, *w*/*v*) and kept in the dark for at least 1 h. The excitation and emission wavelengths were set at 488 and 514 nm, respectively.

### 2.10. Percentage of Adsorbed Protein (AP), Protein Adsorption Content and Composition

AP was measured according to the method described by Liang et al. [26] with slight modifications. Briefly, the emulsion was separated into two phases by centrifugation (16,000 rpm, 1 h) to collect cream phase and aqueous phase at the bottom. After filtration with a 0.45 μm filter, the collected aqueous phase was used to determine the protein concentration of C_SER_; the cream phase was used for protein composition analysis. The AP and protein adsorption content (Γ) were calculated as follows:

AP (%) = (C_INI_ − C_SER_) × 100/C_INI_ (3)

(4) $$\mathrm{Protein}\;\mathrm{adsorption}\;\mathrm{content}\;\left(\Gamma \right)=\frac{C_{\mathrm{INI}}-C_{\mathrm{SER}}}{6\Phi }\times 1-\Phi \times d_{32}$$

where C_INI_ and C_SER_ represent the initial protein concentration of samples used for the emulsion preparation and the protein concentrations of the aqueous phase, respectively; Φ is oil volume fraction; *d*_32_ represents surface mean particle size of emulsion.

The component of adsorbed protein at the interface was analyzed by SDS-PAGE according to the method described by Li et al. [27]. The cream layer was dispersed in protein dissolving solution. The concentrations of the stacking and separating gels were 3% and 12.5%, respectively. The gel was stained with Coomassie Brilliant Blue G-250 and scanned using a computing densitometer (Molecular Imager Chemi DocXRS+, Bio-Rad, Carlsbad, CA, USA).

### 2.11. Statistical Analysis

All experiments were repeated three times, and the experimental data were expressed as means ± standard deviation. The Origin2016 software was used for both analyzing the data (Diffusion rate constant *K*_dif_) and drawing the graphs. The statistical analysis was performed using SPSS 22 software. In order to test the significant differences of results between different groups, Duncan test was used for one-way analysis of variance (ANOVA). Differences were considered statistically significant at *p* < 0.05.

## 3. Results

### 3.1. Complexation Behavior of PAs with DS and Recovery of PAs

**Figure 1:**
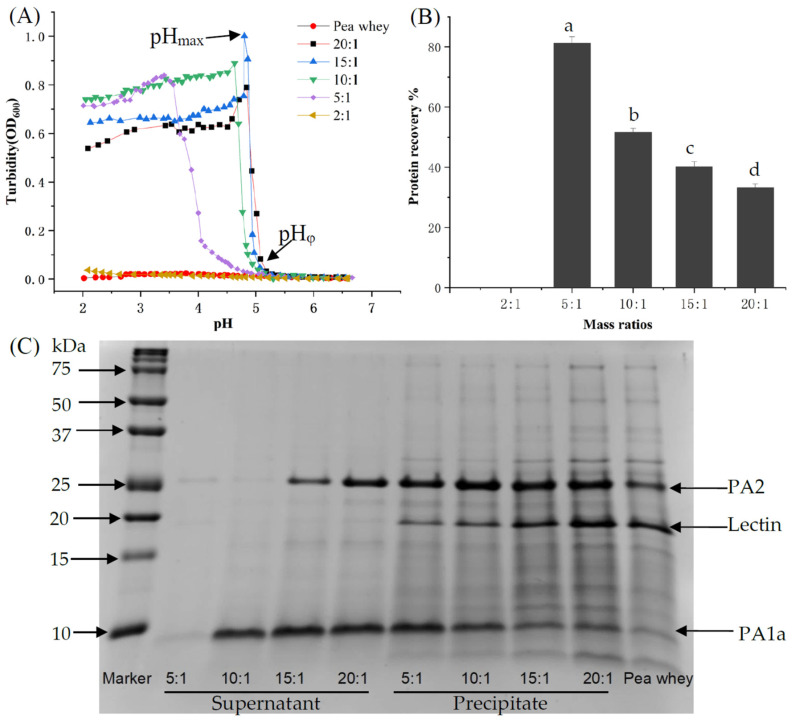
(**A**) Turbidity, (**B**) protein recovery, and (**C**) SDS-PAGE of the PA/DS complexation system. Different letters between different groups indicate significant differences (*p* < 0.05).

Pea whey is a complex system containing large amounts of proteins, oligosaccharides, salt ions, free amino acids and other small molecules. To recover the proteins of pea whey, the complexation behavior of PAs with DS was firstly studied, as shown in Figure 1. Figure 1A shows the changes in turbidity at different pH and mass ratios (*r*). The turbidity of the pea whey was close to zero at pH 6.5–2.0, indicating the good solubility of PAs under acidic conditions. After complexation with DS, the turbidity of pea whey underwent a phase separation process from insoluble complexes to complexes with maximum turbidity, corresponding to pH_φ_ (the onset of insoluble complexes) and pH_max_ (the maximum complexation), respectively [28,29]. The above critical pH values shifted to lower pH values as the mass ratios decreased, because more positive charges were required to compensate for the increased negative charge of DS molecules [30]. The electroneutral characteristic of complexes at pH_max_ has been confirmed in a previous report [17]. There was negligible turbidity observed for the PA/DS system at *r* = 2:1, indicating that only soluble complexes were formed. The excess amounts of DS inhibited phase separation of PA/DS complexes, since the protein charge cannot neutralize the excess negative charge.

The protein recovery at pH_max_ under different mass ratios is shown in Figure 1B. Protein recovery increased with decreasing mass ratio, and over 80% of proteins were recovered at *r* = 5:1. At *r* = 2:1, the PA/DS system became homogeneous without any phase separation, due to the excess of polysaccharides. After complexation with DS, the recovered proteins in the precipitate, as well as the residual protein in the supernatant were analyzed by SDS-PAGE (Figure 1C). Three PAs, PA2, PA1a, and lectin, were found in the precipitate, regardless of the mass ratio, suggesting that they co-precipitated with the polysaccharides. The residual proteins in the supernatant were mainly consisted of PA2 and PA1a, and both disappeared at low mass ratio (*r* = 5:1). This result further confirmed the high recovery of PAs by complexation with DS. The precipitate obtained at *r* = 5:1 can further form soluble complexes through complexation with DS at *r* = 2:1, which will affect the emulsifying ability of PAs at acidic pH conditions. Thus, the difference in the emulsifying property of PA/DS complexes versus PAs alone was further compared.

### 3.2. Interfacial Adsorption Behavior of PAs and PA/DS Complexes

**Figure 2:**
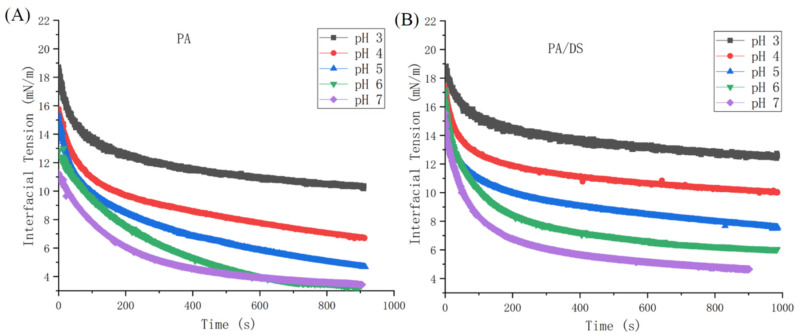
Interfacial tension of PAs (**A**) and PA/DS (**B**) at pH 3–7 as a function of time (s).

The interface adsorption behavior of PA solution and PA/DS complexes at the oil/water interface was investigated using interfacial tension. As shown in Figure 2, with the increase in the adsorption time, the observed interfacial tensions of PA solution and PA/DS complexes showed a decreasing trend, suggesting a progressive adsorption process of PAs or PA/DS complexes to the interface. PA/DS complexes exhibited higher interfacial tension, compared to PAs alone at the same pH condition. This was due to the weak interface adsorption ability of DS. The interfacial tension of PA/DS complexes and PAs alone deceased with increasing pH. This result is consistent with a previous study [31], in which soy whey proteins/soluble soybean polysaccharide mixtures had higher interfacial tension at a low pH. Gharsallaoui et al. [32] found that the protein adsorption to the interface became slower at lower pH conditions. In addition, the strong electrostatic interaction of PAs with DS at pH 3–4 might affect the interfacial adsorption of proteins.

Table 1 shows the diffusion rate constant (*K*_dif_) of PA/DS complexes and PAs alone at different pH values. For PA/DS complexes, the *K*_dif_ decreased at lower pH, suggesting that the protein adsorption process was delayed by the strong interaction of PAs with DS. A similar trend in the diffusion rate with varied pH was reported for β-conglycinin–pectin complexes [33], and soybean protein–soy hull polysaccharide complexes [34]. The formation of homogeneous and condensed protein–polysaccharide electrostatic complexes at low pH might limit the exposure of hydrophobic groups of proteins, thus reducing the surface diffusion and adsorption of proteins. This can be further confirmed by the fact that PAs alone had higher *K*_dif_ than PA/DS at pH 3–5. The *K*_dif_ of PAs alone increased gradually from pH 7 to 5 and then decreased slightly at pH 4 and 3. This was attributed to the strong surface hydrophobicity of PAs around its pI (~pH 4.5–4.8), promoting molecule diffusion and fast adsorption at the oil–water interface. Table 1 also shows that the *K*_dif_ of PA/DS was smaller compared to PAs alone at the same pH (3–5), indicating that the protein molecule diffusion was hindered by the complexation with polysaccharides [35]. However, the effective adsorption of DS at the oil–water interface was actually more conducive to the long-term stability of the emulsion (see Section 3.3), because it can enhance the electrostatic repulsion between oil droplets and the thickness of the interfacial layer.

### 3.3. Emulsifying Ability and Stability of PAs and PA/DS under Acidic pH

#### 3.3.1. Emulsifying Ability

Table 2 shows the change in zeta potential and particle size of emulsions at different pH and heating treatments. The zeta-potentials of PA emulsions were around −16.3 to −30.4 mV at pH 7–5, and became positive at pH 3 and 4. The emulsions stabilized by PA/DS complexes were highly negatively charged, with zeta potentials ranging from −57 to −45 mV. The increase in the zeta potential with decreasing pH was due to the partial charge compensation by positively charged proteins. In addition, there was no significant difference in the zeta potentials between heat-treated and unheated emulsions. The high absolute value of zeta potential is beneficial to the stability of the emulsion. PA emulsions with low absolute potential value (<30 mV) at pH < 6 is more susceptible to flocculation and instability. On the contrary, the high negative charge of DS made the adsorbed PA molecules carry large amounts of negative charges upon the formation of soluble complexes, thus increasing the electrostatic repulsion of emulsion droplets.

Table 3 shows the mean particle size of stabilized emulsions of PAs and PA/DS complexes. The mean particle size of the fresh unheated PA emulsion was less than 1 μm at pH 3, 4, and 7. However, there was a sharp increase in the particle size (over 4 μm) at pH 5 and 6, suggesting that the flocculation of oil droplets occurred. As described in our previous work [17], the pI of lectin and PA2 is 5.31 and 5.16 respectively; thus, the aggregation of both proteins was the largest around pH 5–6. Heat treatment can further cause denaturation and aggregation of proteins, further increasing the oil droplet size. On the contrary, the mean diameter of PA/DS emulsions was smaller than that of PA emulsion, especially at pH 5 and 6 (only ~0.2 μm). These results show that the formation of PA/DS soluble complexes can prevent the protein aggregation (Figure 1), enhancing the emulsifying stability under acidic conditions. Highly charged polysaccharides (carrageenan, dextran sulfate, etc.) can limit the formation of protein-protein aggregates by the higher repulsive force [36]. At pH 3 and 4, more homogeneous and condensed structures of complexes might be favorable to the formation of smaller oil droplets during emulsification. Additionally, the oil droplet size of PA/DS emulsions increased insignificantly (*p* > 0.05) after heat treatment, suggesting the good thermostability of PA/DS complexes to protect proteins from aggregation.

**Figure 3:**
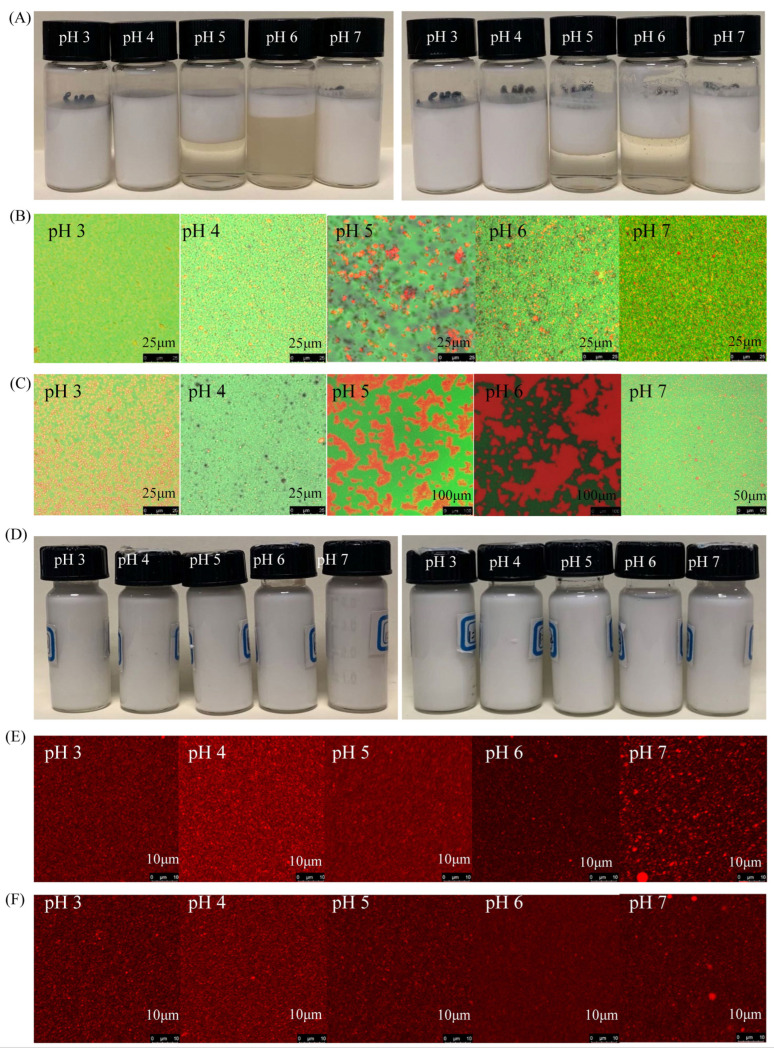
Visual observation and CLSM images of emulsions stabilized by PAs and PA/DS at different pH values. (**A**,**D**) Visual observation of PAs and PA/DS emulsions, respectively (left row, unheated; right row, heated). (**B**,**C**) CLSM images of PAs emulsions with unheated and heated treatments, respectively. (**E**,**F**) CLSM images of PA/DS emulsions with unheated and heated treatments, respectively. For (**E**,**F**), the oil phase is shown so as to clearly capture the changes in the oil droplets.

Figure 3 shows the visual observation and microstructure of emulsions. The fresh PA emulsion was homogeneous without visible flocculation or creaming at pH 3, 4, and 7, while obvious flocculation and creaming of emulsion were observed at pH 5 and 6. Similar results were found for heated emulsions. The CLSM images show the uniform oil droplet structure distribution of the PA emulsion at pH 3, 4, and 7. Conversely, there were large amounts of emulsion droplet aggregates at pH 5 and 6, and the size of aggregates increased upon heating. The observed oil droplets of the PA emulsion at pH 7 were larger than those at pH 3 and 4, which is consistent with the result of particle size results. For PA/DS emulsions, the observed oil droplets of the emulsion were homogeneous, without flocculation or coalescence, indicating good emulsifying stability of PA/DS soluble complexes. Thus, for the fresh emulsion, the emulsifying ability of PAs alone was poor at pH 5 and 6, but it can be modified by complexation with DS through formation of stable complexes.

#### 3.3.2. Emulsifying Stability

**Figure 4:**
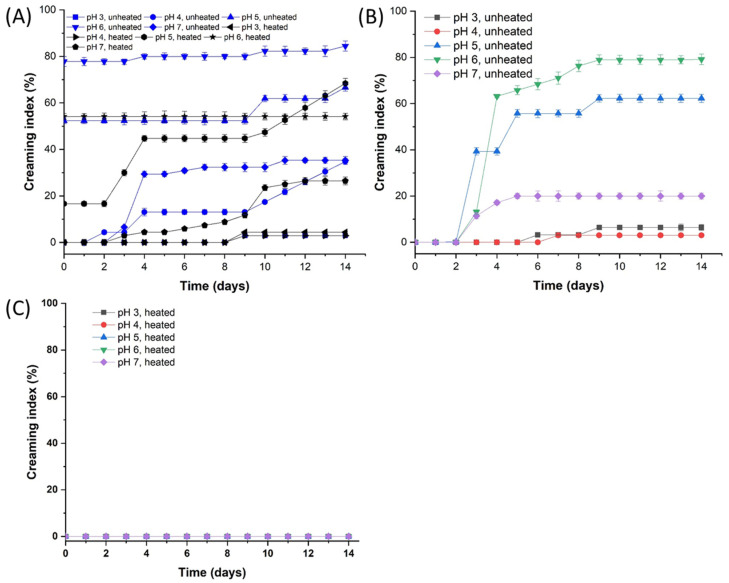
Creaming index (CI) of the O/W emulsions stabilized by PAs alone (**A**) and PA/DS soluble complexes ((**B**) unheated; (**C**) heated) at pH 7–3 upon storage for 14 days.

The emulsifying stability of PAs and PA/DS was characterized by the creaming index (CI) of emulsions under acidic conditions. The CI of PA and PA/DS emulsions over 14 d is shown in Figure 4. High CI values of PA emulsion were observed in pH 5 and 6 samples from 0 to 14 d, and heat treatment did not hinder the creaming behavior, demonstrating the poor emulsifying stability of PAs at these two pH values. The PA emulsions obtained at pH 3, 4, and 7 showed creaming on days 9, 2, and 3, respectively, and their CI reached the maximum on day 14. After heating, the CI decreased to a certain extent depending on the specific pH. However, different degrees of creaming of emulsions still occurred after 14 d of storage. These results reveal that the long-term creaming stability of PA emulsions was poor under acidic pH conditions.

For PA/DS emulsions, the unheated emulsions at pH 7, 6, and 5 was stable in the first 2 days. On days 3–14, the emulsions became unstable and exhibited creaming. Although the unheated fresh emulsions prepared at pH 3 and 4 were stable in the first 5 days, different degrees of flocculation (CI = 6.5%, 3%, respectively) were observed thereafter. The decrease in pH can enhance the creaming stability of PA/DS emulsions and prolong the storage time of the emulsion, which might be attributed to the enhanced interaction between proteins and polysaccharides. Complexing proteins with polysaccharides was reported to be favorable to the improvement in surface activity and emulsion stability of the chitosan/faba bean legumin system [37]. For heated samples, emulsions were stable without flocculation and creaming over 14 d of storage. These results indicate that the combination of complexation with DS and heating facilitated the long-term creaming stability of PA emulsions.

### 3.4. Interfacial Protein Adsorption Behavior and Rheological Properties of Emulsions

#### 3.4.1. Interfacial Protein Adsorption Behavior

As stated above, complexation with DS and heating improved the long-term stability of PA emulsions; however, the role of different proteins in the interface stability of emulsions was still not investigated. Here, the interfacial protein adsorption behavior can clarify the potential synergistic or competitive adsorption behavior of different PAs. Table 4 shows the percentage of interfacial adsorbed proteins (AP) and interfacial protein content (Γ). The AP and Γ of PA emulsions at pH 5 and 6 were not measured due to the creaming behavior (see Figure 3). The AP and Γ of PA emulsions were about two-fold higher than that of PA/DS emulsions (*p* < 0.05). Polysaccharides can adsorb to the interface by interacting with proteins, reducing the approach of proteins through strong electrostatic or spatial steric hindrance surrounding the already adsorbed proteins. It should be noted that the AP and Γ of PA/DS emulsions were the highest at pH 3 and 4. PA/DS complexes had a more condensed and restricted structure at pH 3 and 4 than at other pH values due to stronger electrostatic interaction. This suggests that the spatial steric hindrance of polysaccharides was lowest at these pH values. However, heat treatment markedly improved the AP and Γ of the emulsions, which is consistent with the results of Li et al. [38]. Heat treatment can promote the exposure of the hydrophobic groups in the protein and enhance the adsorption of proteins to the interface.

**Figure 5:**
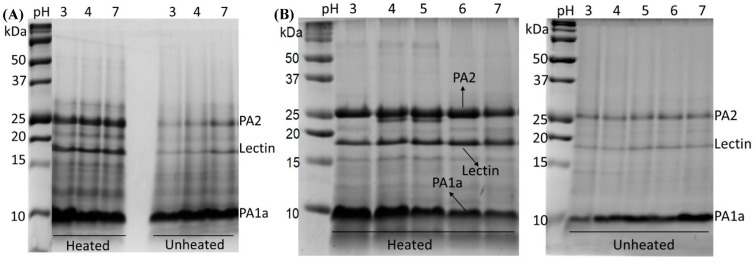
Interfacial protein composition of emulsions stabilized by (**A**) PAs alone and (**B**) PA/DS soluble complexes.

Figure 5 shows the SDS-PAGE results of the absorbed proteins at the oil–water interface. Before heat treatment, the interfacial adsorption proteins of both PAs and PA/DS emulsions were mainly consisted of PA1a, while PA2 and lectin were in less abundant. PA2 was the most abundant protein in the recovered proteins (Figure 1C), but failed to adsorb to the oil–water interface quickly and significantly. The three PAs showed competitive adsorption at the oil–water interface, where PA1a adsorbed preferentially and predominantly compared to the other two PAs. The difference in their molecular properties can explain the difference in interfacial layer composition. PA1a is smaller in molecular weight [39] and less charged than PA2 [27]. As a result, PA1a can adsorb rapidly to the oil–water interface and allow more PA1a to accumulate on the interface, due to the lower electrostatic repulsion between the proteins [19]. However, PA1 had lower emulsifying ability than that of PA2 [20]. PA1a-dominated emulsions, including unheated PAs and PA/DS complexes, showed creaming during the 14 d of storage (Figure 4), which might be due to the relatively weak emulsifying ability of PA1a.

After heat treatment, the abundance of PA2 and lectin increased obviously, as shown in Figure 5. It was reported that heat treatment can increase the protein hydrophobicity, which could cause higher surface activity, especially in the initial adsorption phase [40]. The effective adsorption of PA2 and lectin enhanced the emulsifying ability of PAs, since they had higher emulsifying ability than PA1 [20]. PA2 and lectin contain less cysteine residues compared to PA1a [8]. Thus, their chains are more flexible and are able to generate conformational changes and rearrangement at the interface [41]. Therefore, heat treatment promoted adsorption and rearrangement of PA2 and lectin at the oil–water interface by enhancing the surface hydrophobicity and chain flexibility of them. However, in the absence of polysaccharide, heat-induced denaturation of protein as well as reduction of electrostatic repulsion between protein molecules together resulted in the aggregation of PA2 and lectin under acidic conditions, causing the instability of PA emulsions (Figure 4A). In the presence of DS, the formation of protein–protein aggregates was inhibited by high repulsive force, and the effective adsorption of soluble PA2 and lectin on the interface remained the long-term stability of PA/DS emulsions.

#### 3.4.2. Rheological Properties of Emulsions

**Figure 6:**
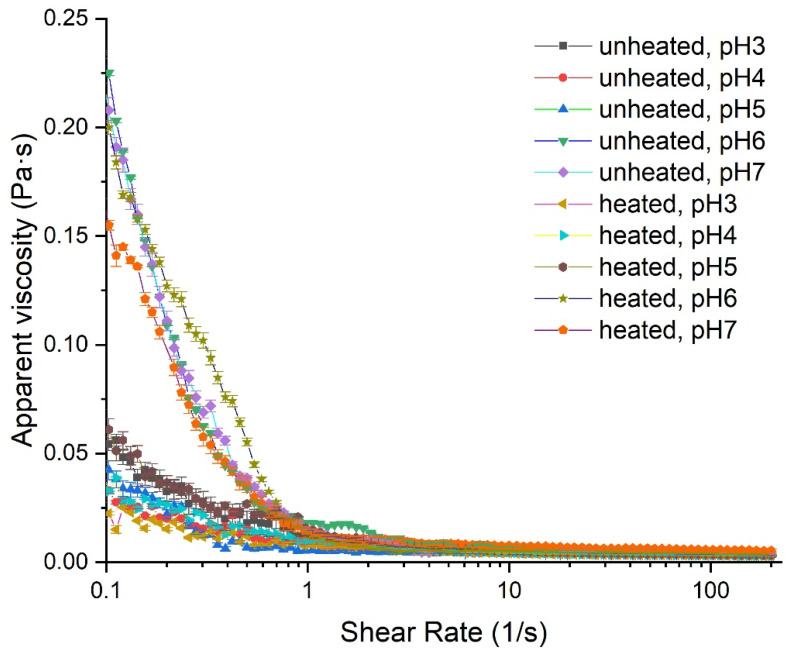
Effect of pH and heat treatment on the apparent viscosity of emulsions stabilized by PA/DS soluble complexes.

The rheological properties of emulsions might affect the emulsifying stability of proteins because polysaccharides are commonly used as thickening agents to enhance the viscosity of the aqueous phase and thereby to mitigate instability mechanisms [42]. Figure 6 shows the apparent viscosity of the PA/DS emulsions as a function of shear rate. The apparent viscosity of PA emulsions at different pH was not investigated due to the creaming phase separation of PA emulsions at pH 5 and 6. A shear-thinning behavior of PA/DS emulsions with increased shear rate was observed, which was due to the deformation of emulsion structure and the breakdown of oil droplets [43]. Damage to the polysaccharide network structure in the continuous phase at high shear rates also contributed to this behavior. In addition, the apparent viscosities of emulsions at pH 7 and 6 were significantly higher than that at pH 5–3. Negatively charged polysaccharides have an extended conformation around neutral pH values, which can enhance continuous phase viscosity of the emulsions [44]. With the decrease in the pH, the enhanced electrostatic interaction between DS and PAs resulted in a condensed structure, thus lowering the viscosity of the emulsions. The high viscosity of the emulsions at pH 7 and 6 seemed to be unfavorable to the long-term stability of PA/DS emulsions (see Figure 4B).

Figure 6 also shows that the apparent viscosity of the emulsions decreased slightly after heat treatment. Similar changes in the apparent viscosity of emulsions at high temperatures have been reported by many researchers [42,45]. In addition, the viscosity of the emulsion is positively correlated with flocculation [46]. It should be noted that the flow curves differed from the previously reported type linear characteristics of protein emulsion systems [47,48]. A similar trend in apparent viscosity was found by Xiang et al. [49] for reconstituted skimmed milk samples. The authors attributed this behavior to the breakdown and restructuring of linkages between protein molecules or between the protein and stabilizer. For the PA/DS emulsion, the linkage between PAs and DS was weak at high pH (6 and 7), due to weak intermolecular interaction; and this linkage became stronger at low pH (3 and 4) due to strong electrostatic attraction between negatively charged DS and positively charged PAs. When the power law model was used to fit the flow curves, the linear coefficient increased with decreasing pH from 7 to 3 (from 0.909 to 0.944), indicating the presence of strong intermolecular interactions at low pH. Heat treatment, as well as the strong intermolecular interactions at low pH, reduced the viscosity of the PA/DS emulsions at pH ≤ 5 and enhanced the resistance to oil droplet flocculation.

## 4. Conclusions

In summary, strong electrostatic complexation occurred between PAs and DS, and the largest amounts of insoluble complexes occurred at *r* = 5:1 and pH_max_. Only soluble complexes were formed at *r* = 2:1 and pH_max_. Based on this principle, PAs were efficiently recovered (> 80%) at *r* = 5:1 and prepared as PA/DS soluble complexes at *r* = 2:1 to improve their emulsifying ability under acidic conditions. PA/DS soluble complexes exhibited higher interfacial tension and lower adsorption kinetics than PAs at pH 3–5. The emulsions stabilized by PA/DS soluble complexes showed good emulsifying stability at pH 3–7; in contrast, the emulsion stabilized by PAs alone exhibited flocculation and creaming at pH 5 and 6. This was due to the aggregation of lectin and PA2, despite their acid-soluble properties prior to emulsification. Complexation with DS improved the stability of emulsions by forming oil droplets with small particle size and high negative charges. PA2, PA1a, and lectin showed competitive adsorption at the oil–water interface, where PA1a was the dominant interface adsorption protein. However, PA1a-dominated emulsions showed creaming over 14 d of storage due to the weak emulsifying ability of PA1a, compared to PA2 and lectin. Heat treatment enhanced the adsorption of PA2 and lectin at the oil–water interface, and complexation prevented heat-induced aggregation. As a consequence, the combination of complexation and heating fulfilled the long-term stability of the emulsion under acidic conditions. This work provides an efficient method of recovery and utilization of PAs as acidic emulsion stabilizers based on complexation with polysaccharides.

## Figures and Tables

**Table 1:** Adsorption kinetics of PAs and PA/DS at pH 3–7 at the oil–water interface.

pH	Diffusion Rate Constant (*K*_dif_, mNm^−1^s^−1/2^)	
	PA	PA/DS
3	0.5067 ± 0.0064 ^c^	0.3754 ± 0.0043 ^a^
4	0.5304 ± 0.0036 ^d^	0.4579 ± 0.0059 ^b^
5	0.5631 ± 0.0049 ^e^	0.4814 ± 0.0058 ^c^
6	0.4462 ± 0.0034 ^b^	0.6601 ± 0.0052 ^d^
7	0.4226 ± 0.0027 ^a^	0.7203 ± 0.0043 ^e^

Different letters in the same column are significant (*p* < 0.05).

**Table 2:** Effect of pH and heat treatment on the zeta potential (mV) of emulsions stabilized by PAs and PA/DS.

Samples	Conditions	pH 3	pH 4	pH 5	pH 6	pH 7
PAs	Unheated	+43.54 ± 0.49 ^a^	+26.87 ± 0.21 ^b^	−16.30 ± 0.53 ^c^	−25.43 ± 0.49 ^d^	−30.42 ± 0.34 ^e^
	Heated	+40.87 ± 0.60 ^a^	+25.53 ± 0.25 ^b^	−15.34 ± 0.34 ^c^	−23.02 ± 0.42 ^d^	−32.57 ± 0.61 ^e^
PA/DS	Unheated	−47.80 ± 1.28 ^a^	−48.80 ± 0.14 ^a^	−53.77 ± 0.71 ^b^	−55.03 ± 1.10 ^b^	−55.90 ± 0.20 ^b^
	Heated	−45.10 ± 0.40 ^a^	−50.13 ± 0.31 ^b^	−53.35 ± 0.35 ^c^	−55.70 ± 0.73 ^d^	−57.23 ± 0.66 ^e^

Different letters in the same row indicate significant differences (*p* < 0.05).

**Table 3:** Effect of pH and heat treatment on particle size (d_4, 3_, μm) of emulsions stabilized by PAs alone and PA/DS soluble complexes.

Samples	Conditions	pH 3	pH 4	pH 5	pH 6	pH 7
PAs	Unheated	0.332 ± 0.025 ^a^	0.456 ± 0.046 ^a^	4.080 ± 0.070 ^c^	4.175 ± 0.045 ^c^	0.928 ± 0.065 ^b^
	Heated	0.302 ± 0.015 ^a^	0.308 ± 0.045 ^a^	10.315 ± 0.375 ^c^	15.060 ± 0.080 ^d^	1.036 ± 0.093 ^b^
PA/DS	Unheated	0.210 ± 0.022 ^a,b^	0.268 ± 0.003 ^a,b^	0.168 ± 0.010 ^a^	0.194 ± 0.013 ^a^	0.463 ± 0.031 ^b^
	Heated	0.229 ± 0.034 ^a^	0.276 ± 0.009 ^a^	0.213 ± 0.041 ^a^	0.213 ± 0.009 ^a^	0.448 ± 0.004 ^b^

Different letters in the same row indicate significant differences (*p* < 0.05).

**Table 4:** Protein adsorption rate (AP) and interfacial protein content (Γ) of emulsions stabilized by PAs alone and PA/DS soluble complexes.

Samples	pH	Unheated		Heated	
		AP (%)	Γ (mg/m^2^)	AP (%)	Γ (mg/m^2^)
PAs	3	58.18 ± 0.69 ^b^	0.56 ± 0.03 ^a^	76.81 ± 1.22 ^b^	1.35 ± 0.02 ^b^
	4	42.00 ± 2.96 ^a^	0.53 ± 0.00 ^a^	72.87 ± 0.02 ^a^	1.17 ± 0.06 ^a^
	7	69.16 ± 0.62 ^c^	0.84 ± 0.01 ^b^	70.08 ± 0.40 ^a^	1.22 ± 0.03 ^a b^
PA/DS	3	25.59 ± 0.05 ^c^	0.47 ± 0.00 ^c^	31.06 ± 0.85 ^b,c^	0.58 ± 0.02 ^c^
	4	32.13 ± 1.01 ^d^	0.87 ± 0.03 ^d^	33.61 ± 0.48 ^c^	0.86 ± 0.01 ^d^
	5	19.06 ± 1.17 ^a^	0.25 ± 0.03 ^a^	20.07 ± 1.22 ^a^	0.34 ± 0.02 ^a^
	6	22.00 ± 0.58 ^b^	0.35 ± 0.01 ^b^	28.62 ± 0.50 ^b^	0.47 ± 0.01 ^b^
	7	19.38 ± 0.85 ^a^	0.26 ± 0.01 ^b^	32.29 ± 0.27 ^c^	0.80 ± 0.00 ^b^

Different letters in the same column indicate significant differences (*p* < 0.05).

## Data Availability

The data used to support the findings of this study can be made available by the corresponding author upon request.
